# The FDA approved PI3K inhibitor GDC‐0941 enhances *in vitro* the anti‐neoplastic efficacy of Axitinib against c‐myc‐amplified high‐risk medulloblastoma

**DOI:** 10.1111/jcmm.13489

**Published:** 2018-01-29

**Authors:** Michael Ehrhardt, Rogerio B. Craveiro, Julia Velz, Martin Olschewski, Anna Casati, Stefan Schönberger, Torsten Pietsch, Dagmar Dilloo

**Affiliations:** ^1^ Department of Pediatric Hematology and Oncology Center for Pediatrics University of Bonn Medical Center Bonn Germany; ^2^ Department of Neuropathology University of Bonn Bonn Germany

**Keywords:** medulloblastoma, Axitinib, GDC‐0941, targeted therapy, multi‐kinase inhibitor, PI3K inhibitor

## Abstract

Aberrant receptor kinase signalling and tumour neovascularization are hallmarks of medulloblastoma development and are both considered valuable therapeutic targets. In addition to VEGFR1/2, expression of PDGFR α/β in particular has been documented as characteristic of metastatic disease correlating with poor prognosis. Therefore, we have been suggested that the clinically approved multi‐kinase angiogenesis inhibitor Axitinib, which specifically targets these kinases, might constitute a promising option for medulloblastoma treatment. Indeed, our results delineate anti‐neoplastic activity of Axitinib in medulloblastoma cell lines modelling the most aggressive *c‐myc‐*amplified Non‐WNT/Non‐SHH and SHH‐*TP53*‐mutated tumours. Exposure of medulloblastoma cell lines to Axitinib results in marked inhibition of proliferation and profound induction of cell death. The differential efficacy of Axitinib is in line with target expression of medulloblastoma cells identifying VEGFR 1/2, PDGFR α/β and c‐kit as potential markers for drug application. The high specificity of Axitinib and the consequential low impact on the haematopoietic and immune system render this drug ideal multi‐modal treatment approaches. In this context, we demonstrate that the clinically available PI3K inhibitor GDC‐0941 enhances the anti‐neoplastic efficacy of Axitinib against *c‐myc‐*amplified medulloblastoma. Our findings provide a rational to further evaluate Axitinib alone and in combination with other therapeutic agents for the treatment of most aggressive medulloblastoma subtypes.

## Introduction

Medulloblastoma is the most common malignant brain tumour of childhood. Despite current intensive therapy including a combination of surgery, chemotherapy and radiation, still one‐third of the patients succumb to this disease 1. Thus, novel treatment modalities are urgently needed.

Signalling *via* vascular endothelial growth factor receptor VEGFR 1/2, platelet‐derived growth factor receptor α/β (PDGFR α/β) and c‐kit is critical for medulloblastoma development and progression 2, 3, 4, 5, 6, 7. Axitinib, a potent angiogenesis inhibitor specifically targets these kinases with high affinity 8.

Axitinib has been approved by the Food and Drug Administration for the treatment of advanced renal cell carcinoma 8. Multiple phase I, II and III studies in adult patients suffering from various cancers including glioblastoma multiforme document the efficacy and good tolerability of Axitinib 9, 10. In comparison with broad‐spectrum multi‐kinase inhibitors such as Sorafenib and Sunitinib, the high specificity of Axitinib and the documented favourable toxicity profile, in particular, with respect to haematologic adverse events, render this drug an ideal candidate for complementation of immunotherapy, chemotherapy and other targeted agents 11, 12, 13, 14.

While the anti‐angiogenic capacity of Axitinib has been extensively delineated, only few reports show that the anti‐tumour activity of Axitinib is also mediated by inhibition of its target kinases VEGFR1‐3, PDGFR α/β and c‐kit expressed by the tumour cells themselves 15, 16, 17, 18, 19, 20. In medulloblastoma, aberrant activation of these receptor tyrosine kinases (RTKs) is considered key to tumour development and progression 2, 3, 4, 6, 7, 21. PDGFR α and β expression, in particular, have been found to be characteristic of metastatic disease and correlates with poor prognosis 5, 6. In a xenograft mouse model, we previously documented tumour regression and prolonged survival following treatment of orthotopic medulloblastoma with the broad spectrum multi‐kinase inhibitors Pazopanib and Sorafenib 22. In comparison with these drugs, Axitinib exhibits an exceedingly low IC50 for the mentioned RTKs such that it recommends itself as a highly attractive agent especially for multi‐modal treatment approaches 8, 23. To date, Axitinib has been successfully incorporated into the treatment regimes of adult malignancies 8, 9. However, studies evaluating its efficacy in paediatric tumour entities are lacking.

Here, we report that Axitinib displays anti‐proliferative, anti‐clonogenic and pro‐apoptotic activity in cell lines modelling the most aggressive *c‐myc‐*amplified Non‐WNT/Non‐SHH and SHH‐TP53‐mutated medulloblastoma variants associated with a very poor prognosis 24, 25, 26, 27, 28, 29, 30. In line with these findings, we demonstrate that Axitinib treatment reduces the phosphorylation state of the signalling molecules AKT and signal transducer and activator of transcription 3 (STAT3) known to be downstream of the targeted kinases VEGFR1/2, PDGFR α/β and c‐kit. Recently, our research group documented *in vitro* and *in vivo* the anti‐neoplastic potential of the phosphoinositid‐3‐kinase (PI3K) inhibitor GDC‐0941 for medulloblastoma therapy 31. Here, we show that Axitinib in combination with GDC‐0941 displays enhanced cytotoxic and anti‐proliferative efficacy alongside with a complete abrogation of AKT and STAT3 signalling in *c‐myc‐*amplified medulloblastoma. Thus, our data provide a rational for further assessment of Axitinib alone and in combination with other therapeutic approaches such as PI3K inhibitors for medulloblastoma treatment.

## Material and methods

### Reagents and antibodies

Axitinib and GDC‐0941 were obtained from LC Laboratories (Woburn, MA, USA). The primary antibodies pSTAT3 (TYR705, D3A7), STAT3 (124H6), pAKT (Ser473), AKT (11E7) and GAPDH were purchased from Cell Signalling while secondary antibodies were purchased from Dianova (Hamburg, Germany). Carboxyfluoreszein‐succinimidyl ester (CFSE) was purchased from Invitrogen (Carlsbad, CA, USA), while Hoechst 33258 was provided by Sigma‐Aldrich (St. Louis, MO, USA).

### Cell culture

The human medulloblastoma cell lines Daoy (HTB 186) and D283 Med (HTB‐185) were obtained from American Type Culture Collection (ATCC). The medulloblastoma cell line MEB‐Med‐8A was generated by Prof. T. Pietsch. The medulloblastoma cell lines Daoy, D283 Med and MEB‐Med‐8A were maintained in complete medium, namely Dulbecco's modified Eagle's medium (DMEM, PAA), with l‐glutamine supplemented with 1 mM sodium pyruvate (PAA), 1% penicilline/streptomycine (Invitrogen) and 10% foetal bovine serum (FBS, Invitrogen).

### Quantification of cell numbers

MEB‐Med‐8A (7 × 10^5^), D283 Med (5 × 10^5^) and Daoy (3 × 10^5^) cells were seeded in 6‐well cell culture dishes in complete medium. After overnight cultivation, the cells were treated with the respective drug concentration for 24 and 48 hrs. Dimethylsulfoxid (DMSO) served as control. Thereafter, dead and living cells were harvested and resuspended in an equal volume of PBS. Dead cells were identified by trypan blue stain according to the supplier's instructions. Results of microscopic cell count by means of a haemocytometer were confirmed by a flow cytometric analysis. Dead cells were excluded from analysis by Hoechst33258 stain. For each sample, the total number of living cells measured within 100 sec. was determined.

### Cell proliferation and cell death assay

Medulloblastoma cells were stained with CFSE according to the supplier's instructions. MEB‐Med‐8A (7 × 10^5^) and D283 Med (5 × 10^5)^ and Daoy (3 × 10^5^) cells were seeded in 6‐well cell culture dishes in complete medium. After overnight cultivation, the cells were treated with respective concentrations of Axitinib and GDC‐0941 for 48 hrs. Thereafter, floating and attached cells were collected, stained with Hoechst33258 and analysed by flow cytometry. Proliferation was traced by CFSE staining and normalized to the control DMSO, while dead cell was determined by Hoechst33258 staining.

### Colony formation assay

The cell lines Daoy (200 cells/well) and MEB‐Med‐8A (1000 cells/well) were plated in 6‐well cell culture dishes. The cells were allowed to adhere and spread properly for 12 hrs at 37°C. Thereafter, the cells were exposed to the stated concentration of Axitinib. After 48 hrs of exposure, the cells were washed with standard medium to remove any trace of the inhibitor and cultured for another week. Colony numbers and colony size were assessed by IMAGEJ. Particles smaller than 40 pixel^2^ were excluded from the analysis as these represented stain artefacts, cell detritus or non‐proliferating single cells.

### Immunoblotting analysis

The medulloblastoma cell lines Daoy, MEB‐Med‐8A, D283 Med and D341 were exposed to different Axitinib concentrations for 1 and 48 hrs, respectively. Thereafter, the cells were harvested and subsequently lysed in RIPA buffer (Sigma‐Aldrich) containing protease and phosphatase inhibitors (protease inhibitor and PhosSTOP phosphatase inhibitor cocktail; Roche (Rotkreuz, Switzerland)). The lysates were centrifuged 10 min. at 10,000 × *g*, and the protein concentration was determined by means of the Bradford assay. During the entire procedure, cells and lysates, respectively, were kept at 4°C. A total protein concentration of 25 μg derived from medulloblastoma cell lines was separated by SDS‐polyacrylamide gel electrophoresis and transferred to nitrocellulose membranes (Bio‐Rad, Hercules, CA, USA). The membranes were blocked for 1 hr at RT in 1× Tris‐buffered saline containing 0.1% tween‐20 (TBST) supplemented with 5% BSA. Thereafter, the membranes were incubated with the primary antibodies (1/1000) overnight at 4°C and subsequently with the respective secondary antibody (1/10,000) for 1 hr at room temperature. Immunoreactivity was detected by chemiluminescence and quantified by means of a ChemiDoc XRS Imaging System (Bio‐Rad).

### Statistical analysis

The two‐sided Student's *t*‐test was applied to determine statistical significance of differences between groups. *P* < 0.05 (*) was considered as statistically significant. Values stated within text and figures represent mean ± standard deviation.

## Results

### Axitinib reduces the viability of different medulloblastoma cell lines in a time‐ and dose‐dependent manner

**Figure 1:**
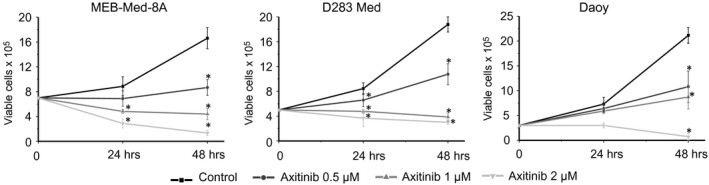
Axitinib reduces the viability of different medulloblatoma cell lines in a time‐ and dose‐dependent manner. The stated medulloblastoma cell lines were seeded and exposed to 0.5, 1 and 2 μM of Axitinib for 48 hrs. Cell viability was assessed by cell count after 24 and 48 hrs. Dead cells were excluded from analysis by trypan blue staining. All values below an asterisk are significantly different from control (*P < 0.05). Each experiment was performed in triplicates and repeated four times.

For our analysis, we chose the paediatric medulloblastoma cell lines MEB‐Med‐8A, D283 Med and Daoy. MEB‐Med‐8A and D283 Med display distinct characteristics of *c‐myc*‐amplified Non‐WNT/Non‐SHH medulloblastoma while the third analysed cell line, Daoy, shows markers of a subgroup of SHH tumours characterized by a mutation of tumoursuppressor protein 53 24, 25, 26, 27, 28, 29, 30. Cells were cultured in the presence of increasing concentrations of Axitinib or the vehicle DMSO as control with *in vitro* concentrations of 0.5 and 1 μM Axitinib corresponding to plasma levels observed in patients (Fig. 1). At 24 hrs, medulloblastoma cell lines have started to proliferate. At this early time, in culture, in the presence of 0.5 and 1 μM Axitinib, cell growth is significantly attenuated in MEB‐Med‐8A, D283 Med in comparison with the untreated control while in Daoy, this effect is observed only after dose escalation to 2 μM Axitinib. In contrast, after 48 hrs, all three investigated cell lines exhibit a significant dose‐dependent reduction of viable cells in comparison with the untreated control with a decrease in viable cell number to 53 ± 11% at 0.5 μM, 27 ± 5% at 1 μM and 8 ± 2% at 2 μM Axitinib in MEB‐Med‐8A and 58 ± 7%, 21 ± 4% and 16 ± 1.5% in D283 Med, respectively. In Daoy, suppression of viable cell number is again less pronounced after 48 hrs at 0.5 and 1 μM Axitinib with 50 ± 17% and 40 ± 11% residual viable cells compared to the untreated control and markedly enhanced cytoreduction at 2 μM Axitinib to 4 ± 3%.

At 48 hrs, significant reduction of viable cells in relation to the number of seeded cells is noted in MEB‐Med‐8A with a cytoreductive effect to 63 ± 11% at 1 μM and 81 ± 5.3% at 2 μM Axitinib and 78 ± 11% and 60 ± 5.3% viable cell numbers in D283 Med. In Daoy, a net loss of viable cells was only detected after 48 hrs exposure to 2 μM Axitinib with only 25 ± 20% of the seeded cells surviving.

### Axitinib interferes with the clonogenicity of medulloblastoma

**Figure 2:**
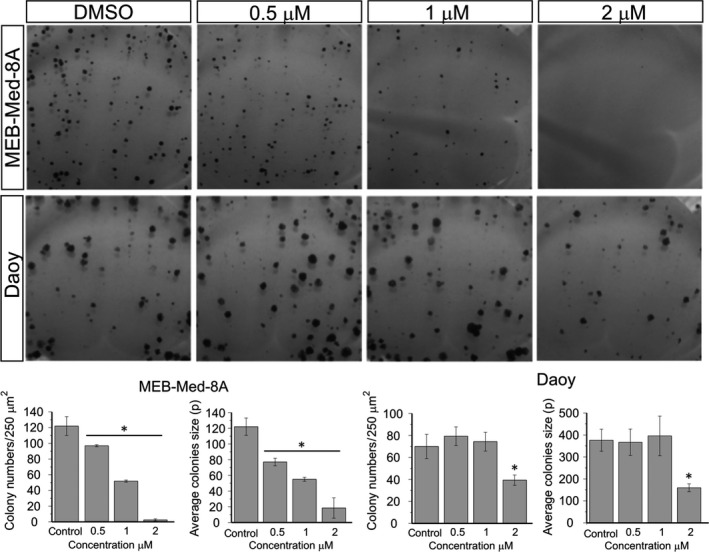
Axitinib interferes with the clonogenicity of medulloblastoma. Daoy and MEB‐Med‐8A cells were exposed to 0.5, 1 and 2 μM Axitinib for 48 hrs. Subsequently, the cells were maintained in standard growth medium for 7 days and colony formation and colony size were assessed. Statistically significant differences from control are marked by an asterisk (*P < 0.05). The data shown represent five independent experiments.

Based on their different group affiliation, the medulloblastoma cell lines MEB‐Med‐8A and Daoy were chosen to assess Axitinib‐induced modulation of colony formation (Fig. 2). In MEB‐Med‐8A, Axitinib reduces colony number and size in a dose‐dependent manner, while in Daoy only the highest concentration exerts a significant inhibitory effect. After exposure to 2 μM Axitinib, only 2 ± 1 colonies grow in MEB‐Med‐8A compared to 122 ± 12 in the DMSO control. In contrast, 39 ± 5 colonies are detectable in Daoy compared to 70 ± 11 residual colonies in the untreated control. Furthermore, Axitinib treatment also leads to profound reduction in colony size with 18 ± 13p^2^ in MEB‐Med‐8A and 158 ± 19p^2^ in Daoy in comparison with 122 ± 11p^2^ and 376 ± 50p^2^ in the respective controls.

### Axitinib alone and in combination with the PI3K inhibitor GDC‐0941 abrogates STAT3 and AKT activity

**Figure 3:**
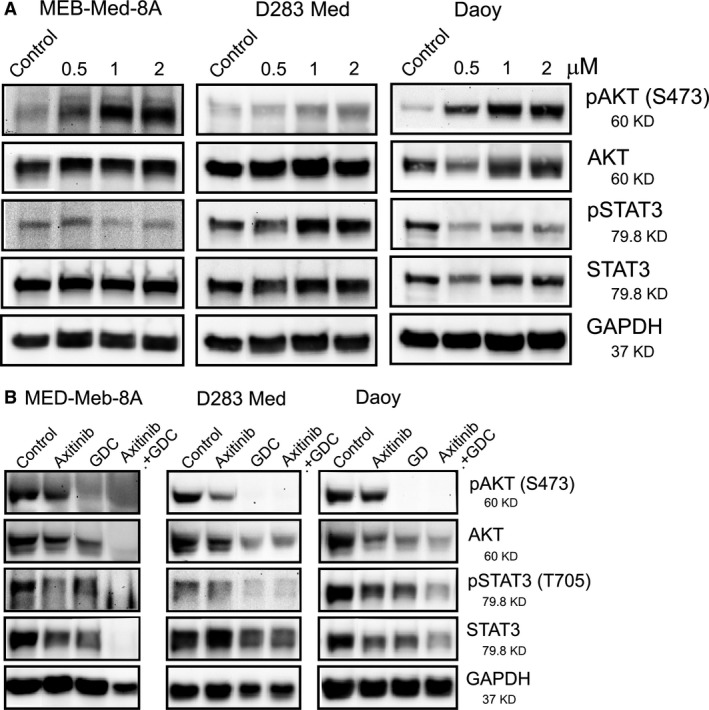
Axitinib alone and in combination with the phosphoinositid‐3‐kinase (PI3K) inhibitor GDC‐0941 affects signal transducer and activator of transcription 3 (STAT3) and AKT signalling. Daoy, MEB‐Med‐8A and D283 Med cells were treated for 1 hr with 0.5, 1 and 2 μM (**A**) and for 48 hrs with 2 μM of Axitinib (**B**), respectively. Furthermore, the stated medulloblastoma cell lines were exposed for 48 hrs to 2 μM Axitinib in the presence of 1 μM GDC‐0941 (**B**). Total protein levels and phosphorylation status of AKT and STAT3 were determined by Western blot. GAPDH served as loading control.

We exposed the medulloblastoma cell lines for 1 hr to 0.5, 1 and 2 μM and determined the activity of the VEGFR 1/2, PDGFR α/β and c‐kit downstream signalling molecules STAT3 and AKT (Fig. 3A). In MEB‐Med‐8A and D283 Med, STAT3 phosphorylation levels remain stable comparable to controls after 1 hr of exposure to 0.5–2 μM Axitinib, while in Daoy, phosphorylation levels are reduced. AKT activity, in contrast, was significantly increased upon drug treatment in all investigated cell lines in a dose‐dependent manner.

Furthermore, 48 hrs treatment with 2 μM Axitinib moderate reduction in STAT3 and AKT phosphorylation is observed in all analysed cell lines (Fig. 3B). Treatment with the PI3K/AKT pathway inhibitor GDC‐0941 alone leads to moderate reduction of STAT3 activity in MEB‐Med‐8A and Daoy and profound impairment in D283 Med. GDC‐0941 also results in complete abrogation of AKT signalling in all cell lines that is therefore not further enhanced by Axitinib. However, with regards to AKT protein levels, combination of Axitinib and GDC‐0941 does have an additive suppressive effect in MEB‐Med‐8A and Daoy. The combination of Axitinib and GDC‐0941 also results in decrease of STAT3 activity in MEB‐Med‐8A and Daoy compared to single drug application. Of note, the suppressive effect on STAT3 protein expression is also additive.

### The PI3K inhibitor GDC‐0941 enhances the anti‐neoplastic efficacy of Axitinib against *c‐myc‐*amplified medulloblastoma

**Figure 4:**
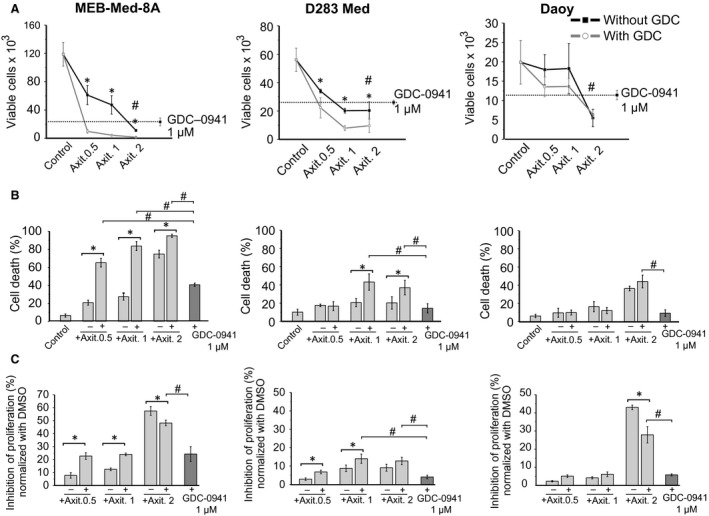
The phosphoinositid‐3‐kinase (PI3K) inhibitor GDC‐0941 enhances the anti‐neoplastic efficacy of Axitinib against *c‐myc‐*amplified medulloblastoma. The cells were treated with the stated concentrations of Axitinib alone and in combination with 1 μM of GDC‐0941. In a combined flow cytometric cell viability/proliferation assay based on a carboxyfluoreszein‐succinimidyl ester (CFSE)/Hoechst 33258 stain, (**A**) the total number of viable cells as number of events acquired in 100 sec. and (**B**) the percentage of dead cells (Hoechst33258^+^ cells) were assessed by flow cytometry after 48 hrs of treatment. (**C**) Inhibition of proliferation, traced by CFSE staining, was normalized to the control dimethylsulfoxid (DMSO) and all stated values differ significantly (*P* < 0.05) from the control DMSO. Statistically significant differences between Axitinib single application and the Axitinib‐GDC combination are marked with an asterisk, while statistically significant differences between GDC‐0941 and the Axitinib‐GDC combination are marked by a hash sign (#*P* < 0.05). The dot line in A represents the number of viable cells upon treatment with 1 μM of GDC‐0941 only for 48 hrs. The data shown represent four independent experiments.

We previously showed that the clinically available PI3K inhibitor GDC‐0941 exerts significant anti‐neoplastic efficacy against medulloblastoma *in vitro* and *in vivo* and displays additive anti‐tumourigenic efficacy with the multi‐kinase inhibitor (MKI) Vandetanib 31, 32. Here, we investigated whether the PI3K inhibitor GDC‐0941 also enhances the pro‐apoptotic and anti‐proliferative activity of Axitinib. For this purpose, we exposed the medulloblastoma lines for 48 hrs to 0.5–2 μM Axitinib in combination with 1 μM GDC‐0941. Cells were analysed and enumerated by flow cytometry following a combined CFSE‐Hoechst33258 stain. The vehicle DMSO served as control (Fig. 4).

In the *c‐myc*‐amplified Non‐WNT/Non‐SHH medulloblastoma lines MEB‐Med‐8A and D283 Med, Axitinib reduces viable cell number in a dose‐dependent manner starting at the lowest concentration of 0.5 μM, while in the SHH‐*TP53‐*mutated cell line Daoy, a significant decrease was only observed at 2 μM, the highest concentration applied. In both the *c‐myc*‐amplified Non‐WNT/Non‐SHH medulloblastoma cell lines, an additive cytoreductive effect of GDC‐0941 is noticeable when combined with 1 and 2 μM Axitinib.

After treatment of MEB‐Med‐8A and D283 Med with 1 μM Axitinib, a decline to 39 ± 5% and 36 ± 5% residual viable cells is observed, respectively, while after exposure to 1 μM GDC‐0941, 19 ± 1.6% and 47 ± 5% cells survive. Compared to either drug alone, the combination of 1 μM Axitinib with 1 μM GDC‐0941 significantly decreases the number of viable cells to 3.6 ± 1**%** in MEB‐Med‐8A and to 14 ± 3% in D283 Med. In MEB‐Med‐8A, cell loss was complete after combined exposure to 2 μM Axitinib and 1 μM GDC‐0941. Yet, in D283 Med, there was no further loss in viable cell number. Daoy, in contrast, is considerably more resistant to Axitinib. Viable Daoy cells are 90 ± 22 and 91 ± 19% at concentrations of at 0.5 μM and 1 μM Axitinib. Notably, cytoreductive activity is documented at 2 μM Axitinib with 30 ± 2.5% cell surviving, which is significantly lower than after exposure to 1 μM GDC‐0941 alone but without any additional effect by the drug combination.

We further dissected the anti‐neoplastic effect of Axitinib with regards to its pro‐apoptotic and anti‐proliferative capacity based on the combined CFSE‐Hoechst33258 stain. There was a substantial cell death effect upon escalation of Axitinib from 1 to 2 μM. After treatment with 2 μM Axitinib, 75 ± 4% of dead cells were detected in MEB‐Med‐8A and 36 ± 3% in Daoy. In MEB‐Med‐8A and Daoy, this exceeds the cell death observed with 1 μM GDC‐0941 resulting in 40 ± 3.5% and 9 ± 4% cell death, respectively. Compared to single drug application, the Axitinib‐GDC‐0941 combination significantly enhances cytotoxicity in MEB‐Med‐8A for all Axitinib concentrations, while in Daoy, dose escalation of Axitinib had the major cytotoxic effect. In D283 Med, increasing the Axitinib dose has no effect on cell death, which remains below 20% as is the case with 1 μM GDC‐0941 alone. However, in combination with GDC‐0941, cytotoxicity is twice as pronounced reaching 43 ± 10% and 37 ± 8% when coadministered with 1 and 2 μM Axitinib, respectively. Moreover, AnnexinV/7ADD staining reveals that Axitinib alone or in combination with GDC‐0941 exerts a pro‐apoptotic effect on all three medulloblastoma cell lines (Fig. S3).

With regards to the anti‐proliferative activity, in MEB‐Med‐8A and Daoy, the most prominent effect is achieved with 2 μM Axitinib alone compared to all other experimental conditions with 57 ± 3.5% and 43 ± 1.5% of growth inhibition, respectively. At lower Axitinib concentrations, the combination with GDC‐0941 augmented the anti‐proliferative effect in MEB‐Med‐8A and D283 Med compared to single Axitinib application at the respective concentration. However, there was no additional effect compared to GDC‐0941 alone in MEB‐Med‐8A and Daoy.

## Discussion

To date, targeted therapies have become an integral element of cancer treatment 33. Multi‐kinase inhibitors with anti‐angiogenic properties also directly impair signal transduction pathways critical for neoplastic transformation and tumour progression. The availability of anti‐cancer drugs with partially redundant target profiles such as Vandetanib, Sorafenib, Sunitinib and Pazopanib is steadily increasing 34. Axitinib stands out from these broad‐spectrum MKI, as it displays one to two log higher affinity to the receptors for VEGFR1‐3, PDGFR α/β and c‐kit with a favourable toxicity profile that renders Axitinib well suited for combination therapy with other targeted agents 8.

While Axitinib is currently under clinical investigation in glioblastoma, this is a first report documenting profound anti‐neoplastic activity in paediatric medulloblastoma cell lines 10. In MEB‐Med‐8A and D283 Med with specific characteristics of the most aggressive *c‐myc‐*amplified Non‐WNT/Non‐SHH medulloblastoma variant 24, 25, 26, 27, 28, 29, Axitinib reduces viable cell number in a dose‐dependent manner with the highest responsiveness in MEB‐Med‐8A. Of the three medulloblastoma cell lines assessed, MEB‐Med‐8A is the only cell line that does not express cancer stem cell markers (Fig. S1). At the highest dose, significant cytoreductive activity is also achieved in Daoy representing the TP53‐mutated subgroup of SHH tumours with extremely poor survival rates 30. The observation that the SHH‐TP53‐mutated cell line—Daoy—is less susceptible to lower doses of Axitinib might also reflect the fact that Daoy differs in its target profile from the myc‐amplified medulloblastoma lines MEB‐Med‐8A and D283 Med with similar VEGFR1‐3, PDGFR α/β receptor but lower levels of c‐kit receptor expression (Table S1).

The profound loss of cell viability in all three MB lines can be attributed to marked pro‐apoptotic and anti‐proliferative activity. Axitinib arrests proliferation in G2/M phase (Fig. S2) and interferes with medulloblastoma clonogenicity regardless of medulloblastoma group affiliation.

In clinical pharmacokinetic studies, there is a high degree of variability in plasma levels even in healthy volunteers. However, although it is well documented that Axitinib is primarily metabolized by CYP3A4/5 and to a lesser extent by CYP1A2, CYP2C19 and UGT1A1, in two meta‐analyses, no association between plasma levels and polymorphisms of these enzymes was found. 35, 36 In clinical anti‐cancer therapy, daily doses range from 10 to 20 mg Axitinib depending on the sensitivity to adverse events and anti‐neoplastic efficacy in the respective patient cohorts. Thus, in renal cell carcinoma patients, plasma levels range between 0.5 and 1 μM following administration of 20 mg of Axitinib 37. Within the range of 0.5 and 1 μM, our *in vitro* experiments proof anti‐neoplastic efficacy against different medulloblastoma cell lines. Morover, *in vivo,* drug accumulation or retention as a result of newly formed dysfunctional tumour vessels might lead to spatial Axitinib concentration in the brain tumour tissue that exceeds the patient plasma concentration 38. Thus, *in vivo,* in addition to direct anti‐tumour effects, modulation of the tumour microenvironment contributes to MKI efficacy. Thus, a recent report on a phase II clinical study in recurrent glioblastoma concludes that Axitinib improves response and progression‐free survival 10. Two additional phase II clinical trials testing the efficacy and safety of Axitinib in patients with glioblastoma are currently under way. (ClinicalTrials.gov Identifier: NCT03291314 and NCT01508117).

In medulloblastoma, we have previously shown in an orthotopic *in vivo* xenograft model that the oral administration of the broad‐spectrum MKI Sorafenib and Pazopanib attenuate tumour growth and prolong survival 22, an effect that was significantly enhanced by the addition of the PI3K inhibitor GDC‐0491 31. Based on the promising *in vitro* cytotoxicity of Axitinib shown here further *in vivo* studies evaluating Axitinib in orthotopic medulloblastoma mouse models are warranted. Pazopanib and Sorafenib also target VEGFR1‐3, PDGFR α/β and c‐kit among other kinases and affect medulloblastoma proliferation and cell survival by inhibition of STAT3 and, to a lesser extend, AKT phosphorylation 22, 39, 40. These observations underline the importance of the respective signalling elements in aberrant RTK activation in medulloblastoma and provide evidence of significant crosstalk between these pathways 31. While prolonged treatment with the highly specific MKI Axitinib impairs both STAT3 as well as AKT activity in MEB‐Med‐8A and Daoy, after 1 hr of drug exposure, transient albeit profound upregulation of AKT activation is observed. This underscores the notion that in medulloblastoma, combined inhibition of both signalling elements STAT3 and AKT might enhance anti‐tumour efficacy.

With acceptable tolerability including limited haemotological side effects, Axitinib is particularly well suited for combination therapy 14, 41. Axitinib also has a favourable profile with respect to the anti‐tumour T cell response with less pronounced inhibitory effects on T cell effector functions compared to other broad‐spectrum MKI 42. In solid tumours including intracerebral neoplastic lesions, Axitinib facilitates a shift from a suppressive to an activated immunologic milieu 43, 44, 45. Thus, MKI‐mediated effects on the haematopoietic and immune system are of particular interest when considering administration in conjunction not only with chemotherapy but also with immunotherapy or other targeted agents 46, 47, 48. Within this context, it has recently been reported that the combination of Axitinib with the anaplastic lymphoma kinase (ALK) inhibitor dalantercept is well tolerated 49. Furthermore, combined inhibition of the mTOR pathway and AKT by Axitinib and Everolimus is momentarily under extensive evaluation in patients with disease progression in various solid cancers (ClinicalTrials.gov Identifier: NCT 01334073). Against this background, we have previously shown that upstream of mTOR/AKT the clinically available PI3K inhibitor GDC‐0941 potently inhibits AKT phosphorylation in high‐risk variants of medulloblastoma and promotes survival in an orthotopic xenograft model 31. Here, we demonstrate that application of GDC‐0941 in combination with Axitinib significantly enhances the anti‐neoplastic efficacy in cell lines harbouring a *c‐myc* amplification compared to treatment with either drug alone. Combination of Axitinib with the AKT inhibitor GDC‐0941 results in near to complete abrogation of viable cells in MEB‐Med‐8A and D283 Med. Corresponding to the enhanced cytotoxicity of the Axitinib‐GDC‐0941 combination, AKT and STAT3 signalling are markedly impaired. While there is a cumulative suppressive effect of Axitinib and GDC‐0941 on STAT3 activity, as expected the specific PI3K inhibitor dominates the inhibitory effect on AKT. The significance of PI3K/AKT signalling has not only been delineated for *c‐myc‐*amplified medulloblastoma but also for cerebellar cells overexpressing *c‐myc* resulting in a similar molecular profile and PI3K dependency 50. Upregulated PI3K signalling is also a feature of cancer stem cells and corresponds to the capacity for self‐renewal in a number of tumour entities including glioblastoma 51. In contrast to the *c‐myc‐*amplified MEB‐Med‐8A and D283 Med medulloblastoma cells, in the SHH‐TP53‐mutated medulloblastoma line Daoy, at the highest dose, the anti‐neoplastic effect of Axitinib exceeds the cytotoxic activity of GDC‐0941 and combination of the two drugs has no additional effect. Of note, across the different medulloblastoma entities, not only the activity of STAT3 and AKT but also protein levels are diminished by the applied inhibitors, which is most likely because of the role of STAT3 and AKT as key regulators of protein expression 52, 53. Our previous study delineates a similar result for the MKI Vandetanib that also targets VEGFR‐2 and 3 but, in addition, the EGFR receptor. In contrast to this study, we have previously documented that the Vandetanib‐GDC‐0941 combination displayed enhanced anti‐neoplastic efficacy compared to single drug application in Daoy 32. This might be because of to the fact that Daoy expresses high levels of the Vandetanib target EGFR but expression of the Axitinib target c‐kit as mentioned above is low. This finding points towards receptor tyrosine expression profiles as biomarker for MKI treatment.

In conclusion, our study demonstrates that Axitinib, a highly specific clinically available inhibitor of angiogenesis, displays marked cytotoxic activity against paediatric medulloblastoma cell lines modelling most aggressive *c‐myc‐*amplified Non‐WNT/Non‐SHH and SHH‐TP53‐mutated tumours. In this setting, marked anti‐proliferative property of Axitinib is complemented by profound cytotoxic capacity. We further show that Axitinib anti‐neoplastic efficacy correlates with the target expression profile on the tumour cells. As the availability of anti‐cancer agents with partially redundant target profiles such as Axitinib, Vandetanib, Sorafenib, Sunitinib and Pazopanib is steadily increasing, biomarkers supporting the choice of MKI are of increasing importance. With regards to multi‐modal treatment approaches, PI3K/AKT pathway inhibition by means of GDC‐0941 enhances the efficacy of Axitinib against *c‐myc‐*amplified medulloblastoma. Our data suggest that Axitinib single treatment or in combination with PI3K inhibitors such as GDC‐0941 might prove a promising strategy to clinically target the most aggressive *c‐myc‐*amplified and SHH‐*TP53*‐mutated medulloblastoma subtypes.

## Conflict of interest

The authors declare that they have no conflict of interest.

## Supporting information


**Fig. S1** Expression of the cancer stem cell markers CD24, CD44 and CD133 is absent in MEB‐Med‐8A.

**Fig. S2** Axitinib induces a G2/M‐phase cell cycle arrest in medulloblastoma.

**Fig. S3** Determination of pro‐apoptotic effects of Axitinib in medulloblastoma cells.

**Table S1** Relative expression of Axitinib target proteins in medulloblastoma cell lines (+ = low; ++++ = high)
