# Supplementary figures and images for: The FDA approved PI3K inhibitor GDC‐0941 enhances in vitro the anti‐neoplastic efficacy of Axitinib against c‐myc‐amplified high‐risk medulloblastoma
Source: J Cell Mol Med. 2018 Jan 29;22(4):2153–61. doi: 10.1111/jcmm.13489 (PMC5867109; doi:10.1111/jcmm.13489)

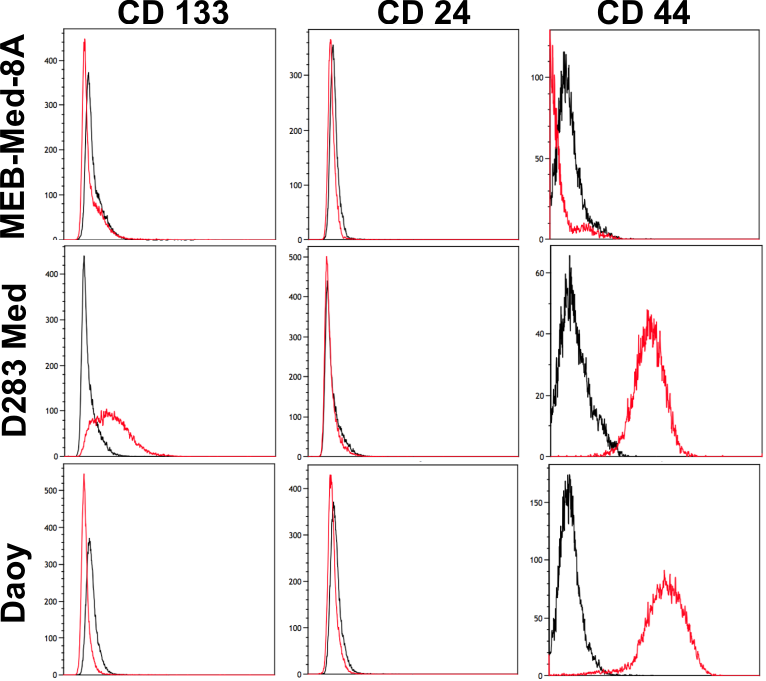

Supplement: Supplementary file 1 — Fig. S1 Expression of the cancer stem cell markers CD24, CD44 and CD133 is absent in MEB‐Med‐8A. [file JCMM-22-2153-s001.tif]

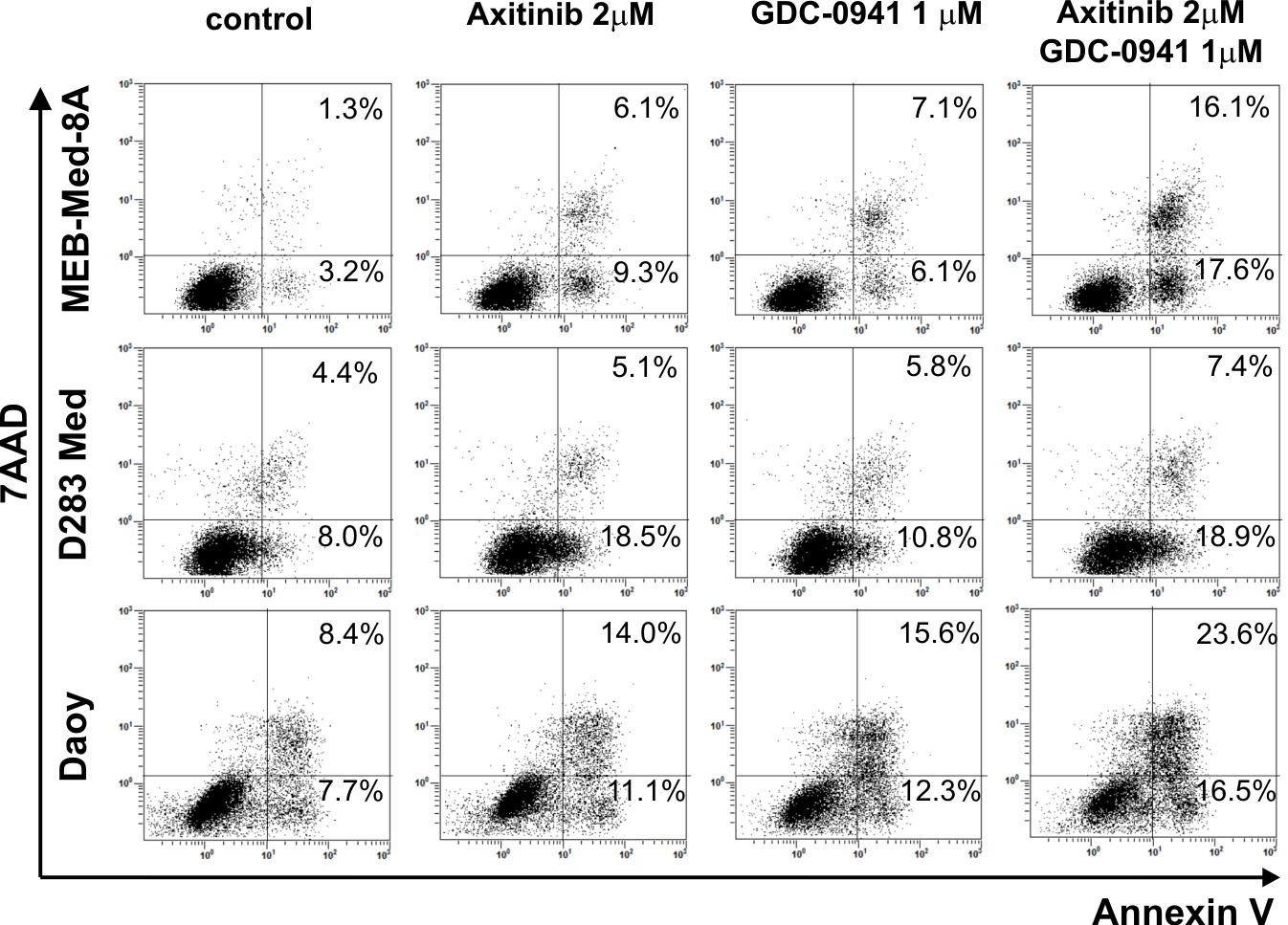

Supplement: Supplementary file 3 — Fig. S3 Determination of pro‐apoptotic effects of Axitinib in medulloblastoma cells. [file JCMM-22-2153-s003.tif]
