# Supplementary material for: The FDA approved PI3K inhibitor GDC‐0941 enhances in vitro the anti‐neoplastic efficacy of Axitinib against c‐myc‐amplified high‐risk medulloblastoma
Source: J Cell Mol Med. 2018 Jan 29;22(4):2153–61. doi: 10.1111/jcmm.13489 (PMC5867109; doi:10.1111/jcmm.13489)
Supplement: Supplementary file 4 — Table S1 Relative expression of Axitinib target proteins in medulloblastoma cell lines (+ = low; ++++ = high) [file JCMM-22-2153-s004.doc]

Supplementary Table 1: Relative expression of Axitinib target proteins in medulloblastoma cell lines (+ = low; ++++ = high)

| **Receptor** | **MEB-Med-8a** | **D283 Med** | **Daoy** |
| --- | --- | --- | --- |
| VEGFR-1 | +++ | +++ | + |
| VEGFR-2 | + | +++ | +++ |
| VEGFR-3 | ++++ | +++ | +++ |
| PDGFRα | +++ | ++++ | +++ |
| PDGFRβ | +++ | +++ | +++ |
| c-KIT | ++++ | ++++ | + |
